# Osteoinductive activity of insulin-functionalized cell culture surfaces obtained using diazonium chemistry

**DOI:** 10.3389/fchem.2014.00117

**Published:** 2015-01-13

**Authors:** Anna Mikulska, Joanna Filipowska, Anna M. Osyczka, Maria Nowakowska, Krzysztof Szczubiałka

**Affiliations:** ^1^Nanotechnology of Polymers and Biomaterials, Faculty of Chemistry, Jagiellonian UniversityKraków, Poland; ^2^Department of Biology and Cell Imaging, Faculty of Biology and Earth Sciences, Jagiellonian UniversityKraków, Poland

**Keywords:** diazoresin, pectin, insulin, bone morphogenetic protein 2, alkaline phosphatase, osteogenesis, human mesenchymal stem cells, cell culture surfaces

## Abstract

Polymeric surfaces suitable for cell culture (DR/Pec) were constructed from diazoresin (DR) and pectin (Pec) in a form of ultrathin films using the layer-by-layer (LbL) technique. The surfaces were functionalized with insulin using diazonium chemistry. Such functionalized surfaces were used to culture human mesenchymal stem cells (hMSCs) to assess their suitability for bone tissue engineering and regeneration. The activity of insulin immobilized on the surfaces (DR/Pec/Ins) was compared to that of insulin dissolved in the culture medium. Human MSC grown on insulin-immobilized DR/Pec surfaces displayed increased proliferation and higher osteogenic activity. The latter was determined by means of alkaline phosphatase (ALP) activity, which increases at early stages of osteoblasts differentiation. Insulin dissolved in the culture medium did not stimulate cell proliferation and its osteogenic activity was significantly lower. Addition of recombinant human bone morphogenetic protein 2 (rhBMP-2) to the culture medium further increased ALP activity in hMSCs indicating additive osteogenic action of immobilized insulin and rhBMP-2.

## Introduction

Recent trends in the biomaterial engineering include modifications of biomaterial surfaces with biological compounds to turn bioinert materials into bioactive ones, providing not only favorable physicochemical and mechanical properties, but also biological activity. Surface functionalization can be done with diazonium compounds, which is a fast, simple and efficient method of surface modification and it can be carried out in mild conditions. One approach is based on the functionalization of biologically active compounds [e.g., biotin (Dequaire et al., 1999), rabbit and human immunoglobulins (Corgier et al., 2005), or DNA (Corgier et al., 2007)] with diazonium groups which then react with the surface to be modified. Such functionalization of proteins with diazonium groups has been used for decades (Phillips et al., 1965) and particularly extensively studied (Berthelot et al., 2011). The other approach, applied in the present study, relies on the surface modification with diazonium groups. These groups may then react with functional groups of the compounds to be coated with (Vergnol et al., 2013; Le et al., 2014). Both electrochemically (Dequaire et al., 1999; Corgier et al., 2005; Vergnol et al., 2013) and photochemically-driven (Plewa et al., 2011) reactions of diazonium groups may be applied.

Diazonium chemistry can be used to obtain 2D and 3D cell culture surfaces and scaffolds, e.g., for photocrosslinking the ultrathin layers forming the culture surface (Plewa et al., 2011) or for functionalization of preformed scaffold [e.g., phosphonation of poly(D,L-lactic acid) (PDLLA) scaffolds (Mahjoubi et al., 2014)].

In this study we have applied diazonium chemistry to immobilize insulin, an important hormone showing a variety of physiological activities, on cell culture surfaces. The latter were obtained using layer-by-layer deposition of diazoresin (DR), a cationic synthetic polymer, and pectin (Pec), an anionic natural polysaccharide, followed by photochemical crosslinking of the obtained multilayer structure (Plewa et al., 2011). We have previously found that such surfaces stimulate the growth of human mesenchymal stem cells (hMSC) and their osteogenic response as indicated by increased alkaline phosphatase (ALP) activity in these cells. Here, we test whether the osteogenic potential of culture surfaces can be further increased by the immobilization of insulin with a mild photochemical method. We also examine whether such culture surfaces support adult human bone marrow-derived mesenchymal stem cell (hMSC) *in vitro* osteogenic differentiation in the presence of recombinant human bone morphogenetic protein 2 (rhBMP-2). rhBMP-2 is a potent growth factor known to induce bone formation (Schmitt et al., 1999) and it has been used in some specific clinical bone regeneration therapies (Govender et al., 2002; Termaat et al., 2005). It was previously reported that the osteogenic response of hMSCs to rhBMP-2 was enhanced by addition of insulin to culture media (Osyczka and Leboy, 2005). We extend these studies to examine whether the effects of immobilized insulin on hMSC cultures differ from those of insulin dissolved in the culture medium.

## Experimental

### Materials

Pectin (Pec) (degree of esterification 70.2%, Sigma Aldrich), 4-diazodiphenylamine sulfate (DDS, Sigma Aldrich), paraformaldehyde (POCH, Gliwice), zinc chloride (POCH Gliwice), tetraethyl orthosilicate (TEOS, Fluka) were all reagent grade and used as received. Water was distilled twice and deionized using Simplicity Millipore Water Purification System. Insulin from bovine pancreas (Sigma-Aldrich), sodium chloride (POCH Gliwice), sodium tetraborate decahydrate (Fluka), boric acid (>99.5%, Sigma-Aldrich), Brij 35 (Sigma-Aldrich) were used as received.

### Cell culture reagents

Unless stated otherwise, all cell culture reagents (media and sera) were purchased from Life Technologies. Bovine Serum Albumin (BSA), L-proline and sodium pyruvate used to prepare serum-free media were purchased from Sigma Aldrich. rhBMP-2 was purchased from R&D Systems (USA), dissolved as recommended by the manufacturer and used at the final concentration of 100 ng/ml.

### Apparatus

UV–Vis spectra were measured using a HP 8452A diode-array spectrophotometer. IR spectra of the irradiated DR/Pec films and of the films with photoimmobilized insulin were obtained on a Bruker IFS 48 spectrometer. Atomic force microscope (AFM) (Picoforce, Veeco, USA) working in tapping mode was used to characterize the surfaces without and with immobilized insulin in air. Standard silicon cantilevers (Veeco) with nominal spring constant 40 N/m and the tip radius <10 nm were used for all the measurements. Photocrosslinking of the DR/Pec films and photoimmobilization of insulin on their surface were carried out using Rayonet photoreactor equipped with six 8 W lamps with the maximum of emission intensity at 350 nm.

### Preparation of diazoresin

Diazoresin (DR) was synthesized according to the procedure described in the literature (Son et al., 2007). Briefly, 4.71 g of 4-diazodiphenylamine sulfate (DDS) was added to a 50 ml flask containing 16 ml of concentrated H_2_SO_4_. The flask was cooled in an ice-water bath. Then, 0.43 g of paraformaldehyde was added in four portions and the reaction was continued for 4 h at the temperature of 0–5°C. The reaction mixture was poured carefully into 30 ml of ice water. Subsequently, 8.02 g of zinc chloride was added to precipitate DR as a ½ ZnCl_2_ complex. After filtration and drying in vacuum, a yellow-green powder was obtained which was kept in the dark.

### Preparation of self-assembled thin polymer films DR/Pec

Pectin and diazoresin were used to obtain ultrathin polymeric films with the layer-by-layer method. Pectin was dissolved in 0.1 M NaCl at the concentration of 1.0 mg/ml. The concentration of DR aqueous solution was 2.0 mg/ml. Multilayer films composed of six bilayers were assembled on the quartz plates according to the literature procedure (Plewa et al., 2011).

### Photoimmobilization of insulin on the surface of the DR/Pec thin films

Insulin solutions were prepared in the borate buffer (pH 7.5) with nonionic surfactant Brij 35 (at 0.06 mg/ml which is equal to the half of its CMC value), and kept in a glass bottle to avoid insulin aggregation (Sluzky et al., 1992). To photoimmobilize insulin onto DR/Pec surface, the multilayer films were immersed in insulin solutions at selected concentrations (up to 36 μM) and irradiated for 7 min. The DR/Pec films without insulin were irradiated in the borate buffer in the same conditions.

### Determination of the immobilized insulin with Coomassie Brilliant Blue G250

Insulin immobilized onto the polymer surfaces was detected using Coomassie Brilliant Blue G250 (Bio-Rad Co.) as an indicator dye (Türkoǧlu et al., 2007). The dye (100 mg) was dissolved in 50 ml of water-ethanol mixture (5:95 v/v) and then the solution was supplemented with 100 ml of 85% (w/v) phosphoric acid. The obtained solution was diluted with water to a final volume of 1000 ml. The DR/Pec films with immobilized insulin (DR/Pec/Ins) coated onto 1 × 2 cm quartz plates were immersed for 1 h into the solution described above (2 ml/plate). The films were then washed with distilled water, dried in a stream of argon and the amounts of immobilized insulin was determined spectrophotometrically by measuring absorbance at 590 nm (Kim et al., 2000).

### Cell isolation and culture on experimental films

Bone marrow stromal cells were harvested from iliac crest of adult patients (age 19–71, both genders) under Institutional Review Board approved protocol (KBET/17/L/2007). The mononuclear fractions, containing both mesenchymal and hematopoietic progenitors, were isolated as described previously (Osyczka and Leboy, 2005). Primary cells were cultured in tissue culture flasks using alpha-MEM supplemented with 10% pre-selected MSC-qualified fetal bovine serum (MSC-FBS) and 1% antibiotics (penicillin/streptomycin; Invitrogen). Cells were maintained in a humidified atmosphere of 5% CO_2_ at 37°C. Medium was changed twice weekly until a confluent cell monolayer was developed. Secondary cultures partly depleted of hematopoietic cells (i.e., hMSC) were used for the evaluation of experimental films. Briefly, glass plates were coated with experimental films composed of 6 bilayers, with Pec as outermost layer. The films were applied to the wells of the 24-well tissue culture plates, insulin was photoimmobilized on them and the wells were presterilized by irradiation with UV light for 5 min. Primary cells were detached from culture flasks using 0.25% trypsin–EDTA (Invitrogen) and seeded onto the films at the density of 2 × 10^4^ cells/ml/well in the 24-well plates. Cells were then cultured on such culture surfaces for 7 days in alpha-MEM supplemented with 10% MSC-FBS and antibiotics (standard culture medium). Where indicated, osteogenic medium consisting of ascorbate-2-phosphate (AA-2P, at the final concentration of 100 μg/ml) and/or recombinant human BMP-2 (rhBMP-2, at the final concentration of 100 ng/ml) was added instead of standard culture medium. Bovine Serum Albumin (BSA, final concentration of 1.25 mg/ml), sodium pyruvate (final concentration of 200 mg/ml) and L-proline (final concentration of 100 mg/ml) were used in SFM instead of FBS. The experimental setup was identical as described previously for serum-containing medium. The cells were seeded and cultured in SFM starting at day 0.

### Cell viability assay

The number of viable hMSCs was estimated using Cell Titer 96 Aqueous One Solution Cell Proliferation Assay (Promega Corp., Madison, WI). Cells were washed once with PBS and each well of a 24-well plate was covered with 200 μl of a solution prepared as a 1:10 (v/v) dilution of 3-(4,5-dimethylthiazol-2-yl)-5-(3-carboxymethoxyphenyl)-2-(4-sulfophenyl)-2H-tetrazolium inner salt (MTS) and phenazine ethosulfate in phenol red-free alpha-MEM. Cells were then incubated for 30 min at 37°C in a humidified 5% CO_2_ atmosphere. Next, the media from each well were transferred to separate wells in 96-well plate and the absorbance was measured at 490 nm.

### Alkaline Phosphatase activity assay

ALP activity was assayed with the use of Alkaline Phosphatase Assay Kit, Sigma86C. At culture day 7 cells were assayed for ALP activity. Briefly, following MTS test, cells were rinsed 3x with PBS. Then, 200 μl of the cell digestion buffer containing Cell Assay Buffer stock solution, composed of 1.5 M Tris, 1 mM ZnCl_2_, MgCl_2_•6H_2_O, diluted 1:10 in dH_2_O and 1% of Triton X-100. was added to each well in 24-well plate and the cells were kept at 4°C overnight. The following day cells were incubated for 30 min at 37°C. The cell lysates were transferred into clean centrifuge tubes, vortexed and centrifuged. To assay for ALP activity, 900 μl of ALP substrate solution (i.e., 37.1 mg of pNPP in 20 ml of Cell Assay Buffer, prepared as described above, was combined with 100 μl of the cell lysate. Following gentle mixing and incubation for 10 min at room temperature, changes in A_405 nm_ were measured over 6 min at 1-min intervals. The obtained values were next normalized against the number of cells obtained from the cell viability assays (MTS test), as described above.

## Results and discussion

### Immobilization of insulin on DR/Pec films

The main goals of this study was to examine whether insulin can be covalently immobilized on a surface using a mild photochemical method and to verify whether such a surface would support growth and differentiation of hMSC. For this purpose DR/Pec photocrosslinkable films were prepared using a method we developed previously (Plewa et al., 2011). The films were previously shown to support the growth of hMSCs and stimulate their osteogenic response (Plewa et al., 2011).

**Figure 1:**
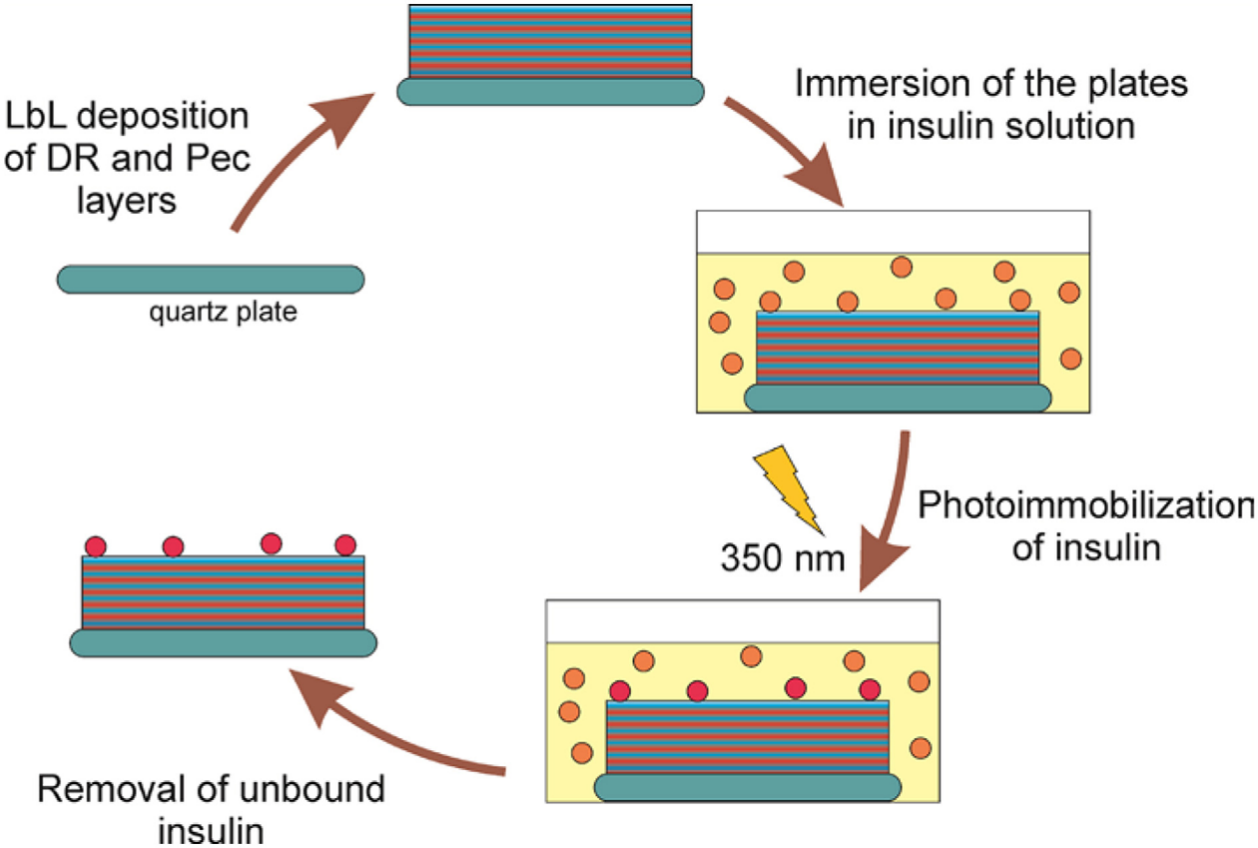
**Fabrication of insulin-functionalized DR/Pec-coated surfaces**.

To attach insulin to the surface of the DR/Pec cell culture surfaces they were layered on the quartz plates, immersed in insulin solutions of different concentrations (i.e., 1.2 nM–36 μM) in a buffer of pH = 7.5), and irradiated with the lamps emitting light with the maximum intensity at λ = 350 nm. This wavelength is absorbed by DR leading to its decomposition and the formation of the reactive phenyl cations, which could react with insulin molecules with the formation of the covalent bonds. The phenyl cations also react with carboxyl Pec groups which leads to the photocrosslinking of the polymeric layers forming the surface (Plewa et al., 2011). The procedure of insulin immobilization is summarized in Figure 1.

Since UV radiation is known to induce chemical and, consequently, functional changes in proteins (Kerwin and Remmele, 2007; Pattison et al., 2012), it was important to examine whether the UV radiation applied did not affect insulin activity. It has been found that irradiation of insulin at 276 nm leads to photodimerization of tyrosine groups within insulin molecules and thus to the formation of covalent insulin dimers (Correia et al., 2012). It can also lead to breaking of disulfide bridges connecting two polypeptide chains of insulin. These changes are reflected in the increase of insulin absorption around 240–285 nm and 285–320 nm (Correia et al., 2012). However, for inducing the photoreaction between DR layers in the films and insulin we used light with comparatively long wavelength, i.e., with maximum intensity at 350 nm. At this wavelength insulin absorption is negligible (insulin does not contain tryptophan (Correia et al., 2012), which absorbs the strongest and at longest wavelengths among the amino acids). Therefore, we expected insulin molecules not to be transformed by the applied radiation. Indeed, we found no changes in the UV spectra of insulin solutions (data not shown) upon irradiation in our particular experimental conditions.

**Figure 2:**
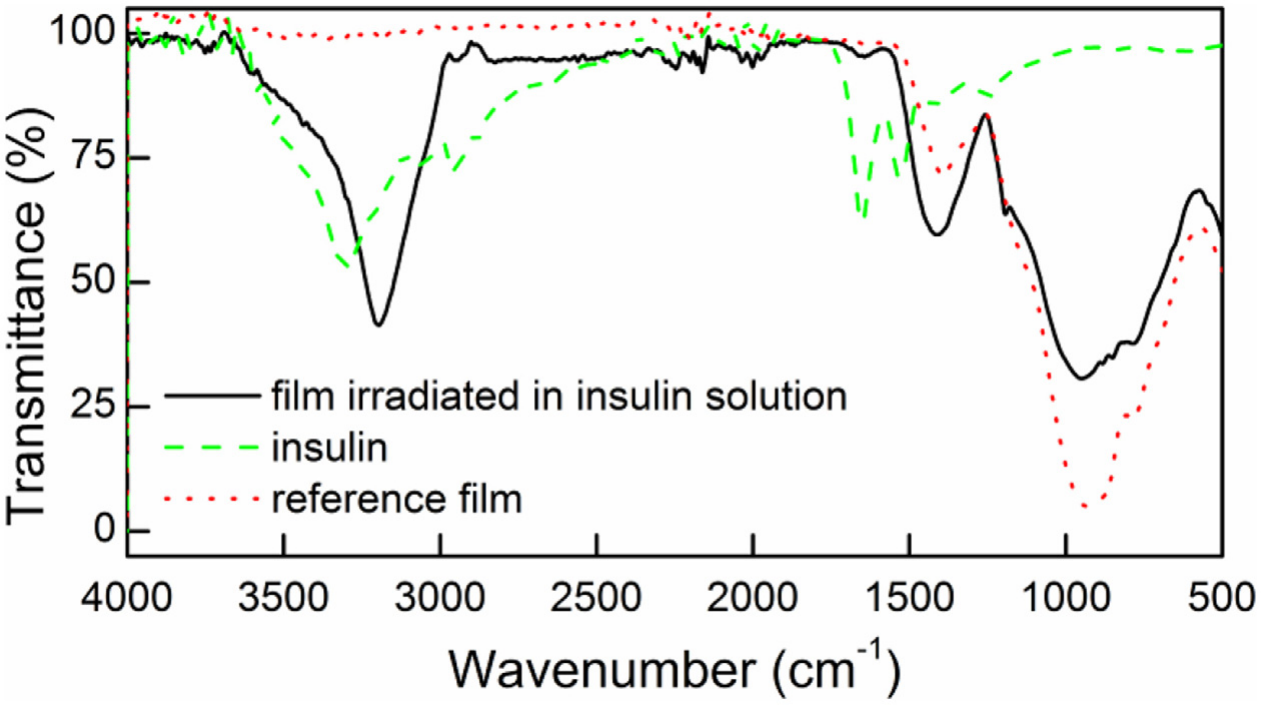
**FTIR spectra for the surfaces irradiated in the presence of insulin (DR/Pec/Ins, solid line), in the absence of insulin (DR/Pec, dotted line), and the spectrum of pure solid insulin (dashed line)**.

To examine whether insulin was covalently attached to the surface of the irradiated films they were analyzed using spectroscopic and microscopic techniques. Figure 2 shows the FTIR spectrum of the surface irradiated in the presence or absence of insulin (DR/Pec/Ins or DR/Pec, respectively), and the spectrum of solid insulin (powder).

In the FTIR spectrum of the surface irradiated in the insulin solution (DR/Pec, Ins, c_Ins_ = 1.8 μM) a weak band at 1654 cm^−1^ and a strong band at 3400–3100 cm^−1^, characteristic of insulin, are present. These bands are absent in the spectrum of the reference (DR/Pec) support, thus confirming the presence of insulin molecules on the surface of the film.

Bradford method of protein detection and quantitation was used to further confirm the presence of immobilized insulin on the film surface. This method is based on the interaction between a protein and Coomassie Brilliant Blue G-250 (CBBG) leading to the complex formation with characteristic absorption at 595 nm (Bradford, 1976).

**Figure 3:**
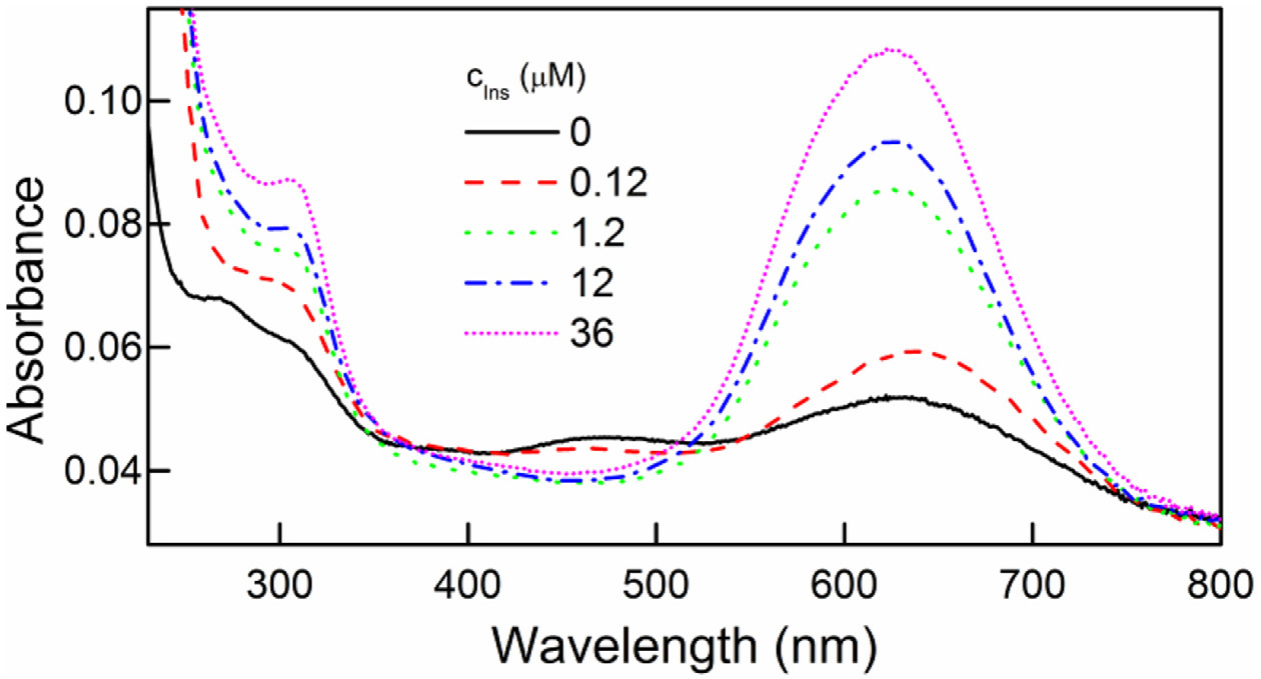
**UV spectra of DR/Pec films irradiated in the absence and in the presence of different concentrations of insulin and then immersed in 0.01% w/v CBBG solution for 24 h**.

We found the intensity of the 595 nm absorption band increased with increasing concentration of insulin solutions in which the films were immersed (Figure 3). This indicated increasing amounts of insulin that were immobilized on the surface of the DR/Pec surfaces.

**Figure 4:**
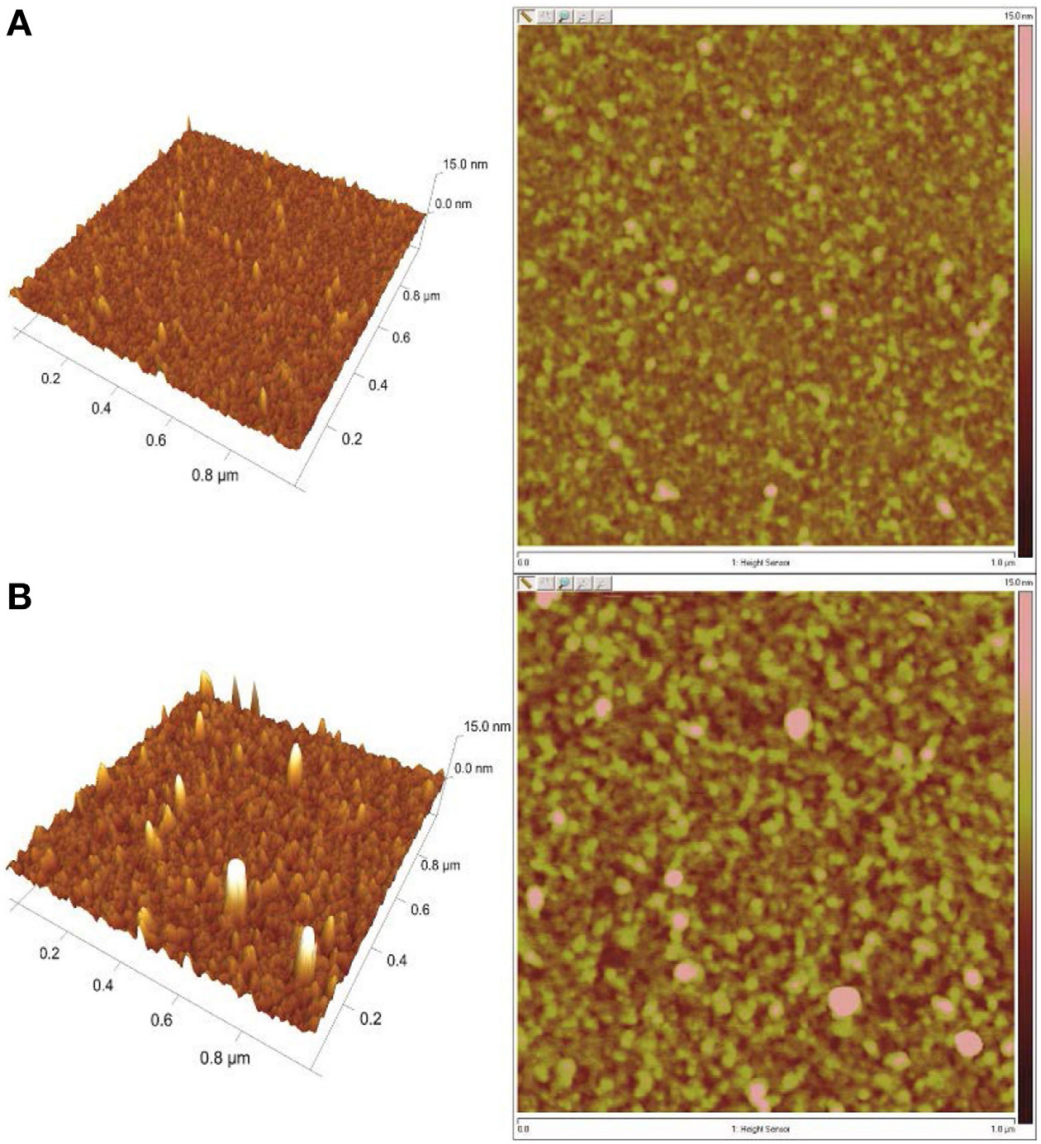
**AFM images of the surfaces irradiated (A) in the absence of insulin (DR/Pec) and (B) in 36 μM insulin solution (DR/Pec/Ins)**.

The presence of insulin on the DR/Pec/Ins surface was also confirmed using AFM microscopy (Figure 4).

The AFM images revealed that the surface of the DR/Pec film irradiated in the absence of insulin is rather smooth with the RMS roughness equal to 0.87 nm while the roughness of the film irradiated in the presence of insulin is much higher (1.74 nm). The increased roughness of this film is due to the several structures seen on the film which can be interpreted as covalently attached insulin molecules. We found several molecules of immobilized insulin per 1 μm^2^. The contact angle of the DR/Pec/Ins surfaces ranged from 20 to 30° vs. 14° for the surfaces not functionalized with insulin. However, we found no clear dependence of the contact angle on the amount of immobilized insulin.

## Human MSC morphology on the insulin-functionalized surfaces (DR/Pec/Ins)

**Figure 5:**
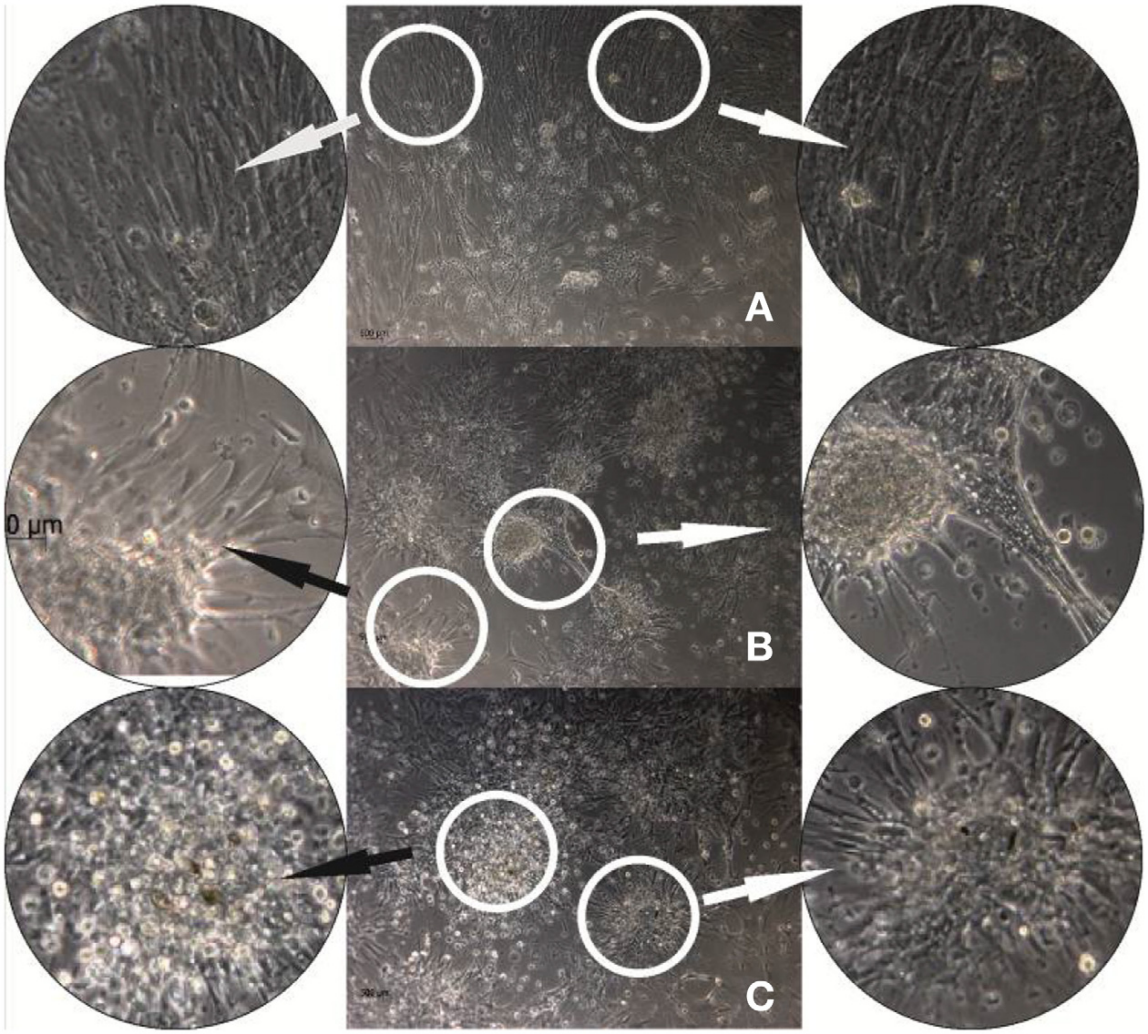
**hMSC cultures grown for 24 h on reference DR/Pec surfaces (A) and on DR/Pec/Ins surfaces obtained by irradiation of DR/Pec films in 4.4 μM (B) and 8.7 μM (C) insulin solution**. The circular photos show 3× magnifications of the most representative fragments of the images.

As we previously showed that DR/Pec surfaces supported growth and differentiation of hMSC, in this work we have extended these studies to examine whether insulin photochemically attached to the DR/Pec surface is able to influence hMSC proliferation and differentiation. Figure 5 shows phase-contrast microscopic images comparing the morphology of hMSC cells cultured for 24 h on the DR/Pec surfaces functionalized with different amounts of insulin and on a reference DR/Pec film.

The images reveal clear differences in the morphology of the cells grown on DR/Pec films with and without immobilized insulin. A homogeneous layer of elongated hMSC cells covered the surface of the DR/Pec film in the absence of insulin, whereas in the presence of insulin hMSC grew in scattered foci with rounded cells in the center and radially protruding elongated and adhering cells. The fraction of rounded cells increased on the film coated with a higher amount of insulin. We also observed that the presence of insulin immobilized on the surface of the supports promotes hMSC proliferation and this was further confirmed by cell viability assay.

## The effect of insulin attached to the DR/Pec film on hMSC viability and ALP activity

### Effect of immobilized insulin on hMSC viability

**Figure 6:**
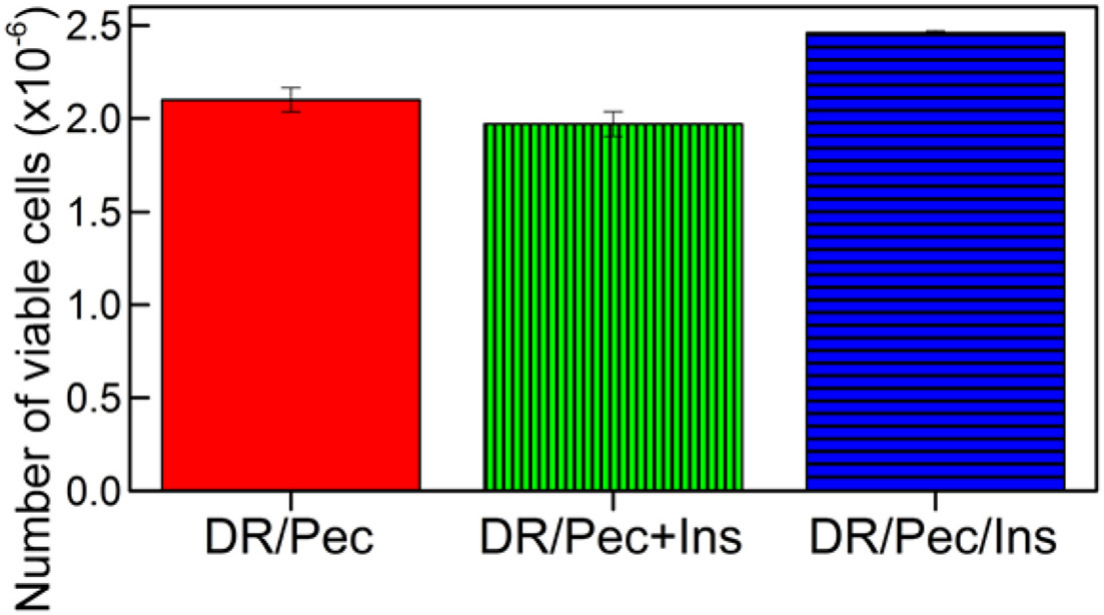
**Number of viable hMSCs cells grown for 7 days on the DR/Pec reference without insulin, DR/Pec surfaces with insulin at 36 μM added to the culture medium (DR/Pec+Ins), and DR/Pec functionalized with insulin (DR/Pec/Ins) by irradiation in 36 μM insulin solution**.

The effect of insulin immobilized on the DR/Pec/Ins surface on hMSC viability was evaluated quantitatively using MTS test. Cells viability was compared to hMSC cultures where insulin was added to the culture medium. We found that the addition of 36 μM insulin to the culture medium does not change the rate of hMSC proliferation (Figure 6). However, the hMSCs proliferation rate was increased by 17% on DR/Pec/Ins surfaces obtained with 36 μM solution of insulin. This suggested the insulin immobilized on the surface can be of higher availability to the cells than insulin simply dissolved in the culture medium. For the sake of comparability, both tests were performed using the same 36 μM insulin solutions.

### Effect of immobilized insulin on osteogenic hMSC response

**Figure 7:**
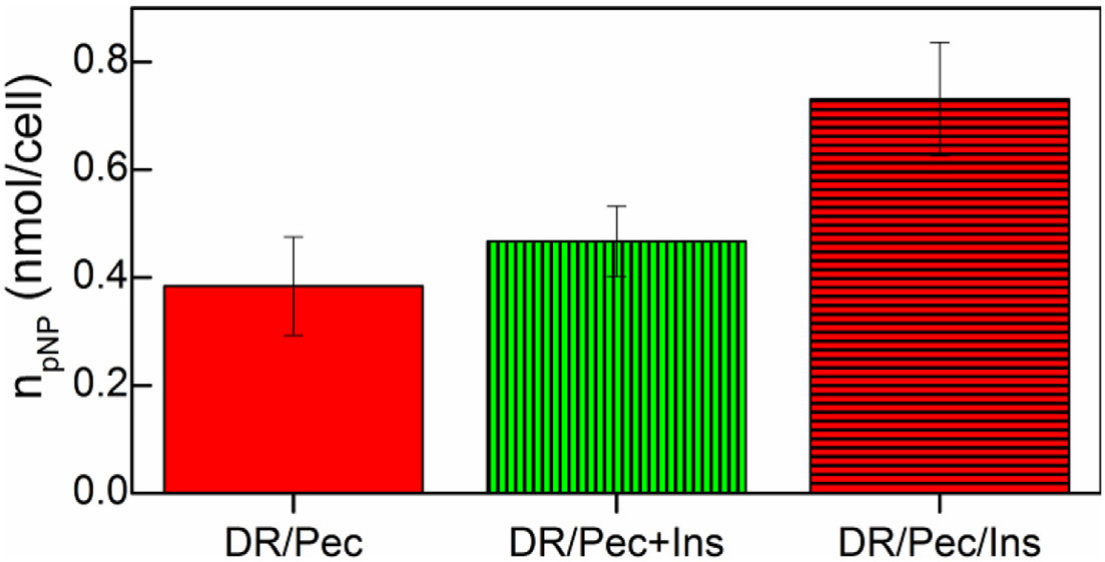
**ALP activity of hMSCs cells grown on the DR/Pec reference surfaces, DR/Pec surfaces with insulin at 36 μM added to the culture medium (DR/Pec+Ins), and DR/Pec functionalized with insulin (DR/Pec/Ins) by irradiation in 36 μM insulin solution**.

The osteogenic response of hMSCs grown on insulin-immobilized DR/Pec culture surfaces was assessed by measuring ALP activity in these cells. We found that insulin-immobilized DR/Pec culture surfaces supported osteogenic response of cultured hMSCs (Figure 7) much stronger than if insulin was supplied in the culture medium.

## Human MSC proliferation and differentiation upon culture on insulin-immobilized DR/Pec surfaces in the presence of rhBMP-2

Earlier reports indicated that rhBMP-2 osteogenic potential can be enhanced in hMSC cultures by addition of insulin to the chemically-defined serum-free culture medium (Osyczka and Leboy, 2005). Thus, we examined whether surface-immobilized insulin supports the osteogenic effect of rhBMP-2. Since the proteins present in the serum-based medium may shield the interactions between insulin and rhBMP-2, hMSCs were cultured in the serum-free medium.

**Figure 8:**
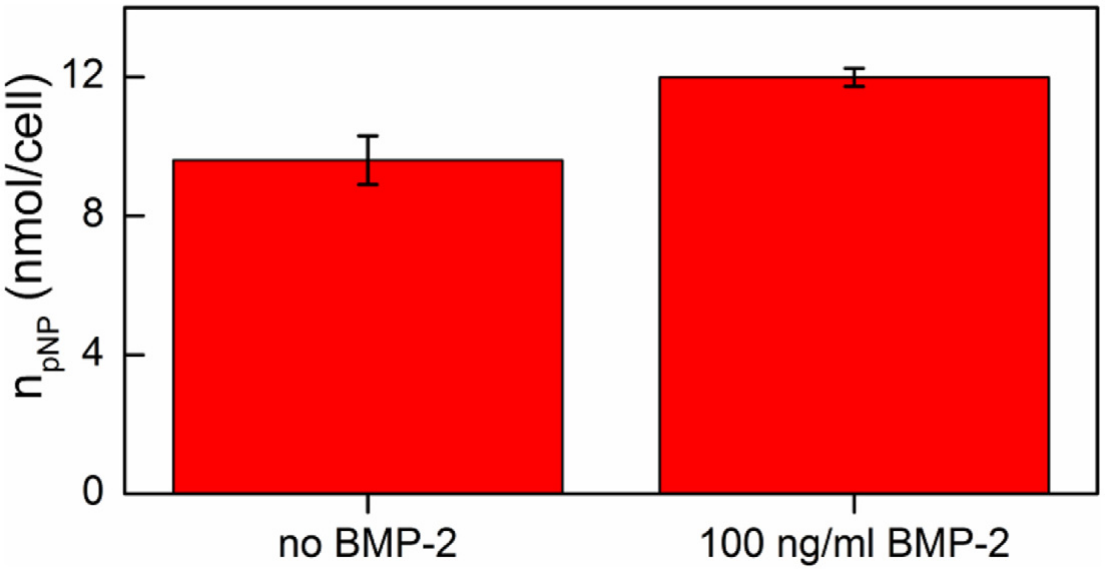
**ALP activity of hMSCs cells grown on DR/Pec/Ins supports in the absence and in the presence of BMP-2 on day 7 of the culture in a serum-free medium**.

The results obtained indicated that addition of 100 ng/ml BMP-2 had no cytotoxic effect on hMSC cells cultured on the DR/Pec/Ins surface (data not shown) while it significantly increased ALP activity in hMSCs (Figure 8).

Thus, one can conclude that the osteogenic effects of insulin immobilized at the surface of DR/Pec surfaces and of rhBMP-2 added to the serum-free medium are additive.

The finding, that the osteogenic action of the DR/Pec surfaces (Plewa et al., 2011) is strengthened by the immobilized insulin (Figure 7) and further increased by the addition of rhBMP-2 (Figure 8), not only confirms previous data of additive positive action of insulin and rhBMP-2 in adult human MSC osteogenesis, but it is also very important from the practical point of view since it implies that the dose of BMP-2 required for bone regeneration may be significantly decreased by its application in combination with DR/Pec/Ins support applied *in situ* in the form of, e.g., films or microspheres. This would allow limiting the high treatment costs and the adverse effects which are due to high BMP-2 doses usually applied in therapy such as osteolysis (Lewandrowski et al., 2007), immune response (Carragee et al., 2011), swelling (Shields et al., 2006), and even life-threatening complications (Carragee et al., 2011). It is suggested that the risk related to the therapeutical administration of recombinant human BMP is 10–50 times higher than previously estimated based on industry-sponsored studies (Carragee et al., 2011). That is why other systems reducing the required dose of BMP-2 have been already proposed (La et al., 2014). The data we obtained may lead to the development of well-cotrollable BMP delivery systems for specific bone-targeting therapies. Therefore, our further studies will aim at immobilizating both molecules and examining their osteogenic potential in adult human bone marrow cultures.

Although the molecular mechanisms of cell response to photoimmobilized or soluble insulin have not been a purpose/focus of this study, our results are in line with the earlier reports by Ito et al. who found that insulin immobilized on different supports (polystyrene, poly(methyl methacrylate), poly(oxyethylene), polyacrylic acid) stimulates the growth of mouse fibroblasts, bovine endothelial cells, and mouse sarcoma cells (Chen et al., 1997a,b; Ito et al., 1997; Li et al., 1997) and shows different or stronger effects than insulin in solution. We suppose that insulin photoimmobilized on the DR/Pec films may have a greater mitogenic activity in hMSC cultures compared to its soluble equivalent, just like in the studies described by Ito. This may be due to inhibition of insulin internalization into the cells and thus immobilized insulin is constantly available for hMSCs and stimulates their growth and differentiation for a longer time compared to its free form. This, however, needs further studies to verify the hypothesis.

### Conflict of interest statement

The authors declare that the research was conducted in the absence of any commercial or financial relationships that could be construed as a potential conflict of interest.
